# Potato Starch Utilization in Ecological Loose-Fill Packaging Materials—Sustainability and Characterization

**DOI:** 10.3390/ma13061390

**Published:** 2020-03-19

**Authors:** Maciej Combrzyński, Arkadiusz Matwijczuk, Agnieszka Wójtowicz, Tomasz Oniszczuk, Dariusz Karcz, Jarosław Szponar, Agnieszka Niemczynowicz, Dariusz Bober, Marcin Mitrus, Karol Kupryaniuk, Mateusz Stasiak, Bohdan Dobrzański, Anna Oniszczuk

**Affiliations:** 1 Department of Thermal Technology and Food Process Engineering, University of Life Sciences in Lublin, Głęboka 31, 20–612 Lublin, Poland; maciej.combrzynski@up.lublin.pl (M.C.); marcin.mitrus@up.lublin.pl (M.M.); karol.kupryaniuk@o2.pl (K.K.); 2 Department of Physics, University of Life Sciences in Lublin, Akademicka 13, 20–950 Lublin, Poland; 3 Department of Analytical Chemistry (C1), Faculty of Chemical Engineering and Technology, Krakow Technical University, Warszawska 24, 31–155 Krakow, Poland; dkarcz@chemia.pk.edu.pl; 4 Toxicology Clinic, Medical University of Lublin, Clinical Department of Toxicology and Cardiology, Stefan Wyszyński Regional Specialist Hospital, Al. Kraśnicka 100, 20–718 Lublin, Poland; 5 Department of Analysis and Differential Equations, University of Warmia and Mazury in Olsztyn, Słoneczna 54, 10–710 Olsztyn, Poland; niemaga@matman.uwm.edu.pl; 6 Interdisciplinary Centre for Computational Modelling, University of Rzeszów, 35–959 Rzeszów, Poland; dbober@ur.edu.pl; 7 Institute of Agrophysics, Polish Academy of Sciences, Doświadczalna 4, 20–290 Lublin, Poland; mstasiak@ipan.lublin.pl; 8 Department of Pomology, Nursery and Enology, University of Life Sciences in Lublin, Głęboka 28, 20–400 Lublin, Poland; bohdan.dobrzanski@up.lublin.pl; 9 Department of Inorganic Chemistry, Medical University of Lublin, Poland, Chodźki 4a, 20–093 Lublin, Poland

**Keywords:** ecological loose-fill packaging materials, extrusion-cooking, foams, process efficiency, cutting force, resistance to compression, FTIR spectra

## Abstract

Biodegradable materials are used in the manufacture of packaging and compostable films and various types of medical products. These have demonstrated high potential in medical applications: cardiac, vascular and orthopaedic conditions in adults as well in children. In our research, the extrusion-cooking technique was used to obtain environmentally friendly loose-fill foams as packaging. Potato starch was the basic raw material. Polyvinyl alcohol was used as an additive in the amount of 1%, 2% and 3% to replace starch. The components were mixed and moistened with water to various initial moisture contents of the blend (17%, 18% and 19%). The processing of starch foams employed the TS-45 single screw extruder-cooker (Gliwice, Poland) with the L/D ratio of 12. The foams were processed with various screw speeds (100 and 130 rpm) and with two types of forming dies (circular and ring die). The extrusion-cooking process efficiency (kg h^−1^) and the energy consumption (kWh kg^−1^) during the processing were also measured. The results showed that the processing efficiency of potato starch foams varied depending on the level of polyvinyl alcohol, the shape of the forming die and the screw speed applied. The analysis of energy consumption, mechanical properties and FTIR analyses demonstrated that the type of the forming die and the initial moisture level had the most significant impact on specific energy demands during the processing of potato starch-based foams.

## 1. Introduction

Rock, soil, wood, fiber and bones of living organisms are natural porous materials [1,2]. The natural environment abounds in porous materials with hierarchically ordered structures of interconnected pores of different lengths. Natural porous materials also still to be discovered and examined, for example, islands and lands formed of volcanic rock (cooled lava). 

First polymeric foamed material was invented in the early 20th century. The first polymer foam was made in the 1930s [3,4] with the use of polystyrene (1931). Polyurethane invented by Dr Otto Bayer was used during the Second World War to substitute rubber or as a protective coating for wood and metal parts. In the 1940s, polyurethane was foamed. The early foam materials were used to protect goods, primarily in the furniture and automotive industries. In the following decades, the opportunities arising from the application of this type of polymers led to a rapid development of manufacturing technologies based on foam materials, including injection molding, extrusion-cooking and other techniques.

Foamed packaging materials are now mainly made of plastics, such as polyurethane, polystyrene or polypropylene [5,6,7,8]. These find application in the protection of cargo during transport and in everyday life, for example, when handling fragile, easily damaged products. Foamed materials offer protection as well as stabilizing items packed for shipment and working as impact absorbers.

The progress in the production and processing of foamed products is a consequence of the development of materials technology with a special focus on polymers. This technology is improving continuously [9]. An example of that is the traditional foamed polystyrene that has been used for the production of packaging for decades. Its application options are now growing, despite the heavy criticism leveled against its management and disposal [8,10,11]. Large-scale production of this type of packaging material results in environmental pollution because of the raw materials of petrochemical origin, with blowing agents being the most harmful of them. For some years now, consumers and legislators have been increasingly pressing on the implementation of environmentally friendly packaging materials [12].

Due to the limited resources of crude oil and mounting environmental concerns, polymer materials (including foamed ones) are more and more often blended with renewable raw materials [13,14]. The research on the production of biodegradable polymers from renewable, natural sources gained momentum at the turn of the 20th century, and the global demand for such materials grows year to year.

An alternative to popular plastics is starch. It is a relatively easily available polysaccharide and one of the most widespread organic substances in nature [15,16,17]. Starch (C_6_H_5_O_10_) is a polysaccharide carbohydrate containing a mixture of amylose and amylopectin and other components such as lipids and proteins [18,19]. Depending on its origin, starch usually contains 15%–30% amylose and 70%–85% amylopectin. Depending on its botanical origin, starch may differ in particle size, shape and morphology. Its physical, chemical and technological properties are largely determined by the content of amylose and the degree of polymerization [18].

Starch has always accompanied and has exerted a great influence on people’s lives [20]. It is contained in human and animal food. It is used as a processing raw material; no bread and bakery manufacture would be possible without it. However, it was not earlier than in the 19th century that starch started to be extracted from various plant raw materials on an industrial scale. Globally, starch is obtained mainly from potato, maize and wheat. Native starch is popular in many industries, however, due to its numerous drawbacks, for example, insolubility in cold water, its use is often quite limited [21]. However, starch lends itself to modification, which helps reduce or even eliminate its flaws. Thermal or pressure-thermal treatments are the simplest methods of physical modification of starch.

Starch polymers are undoubtedly the most widespread group of biodegradable materials available on the market [22,23,24]. Biodegradable materials are used to produce packaging and compostable films as well as various types of products for medicinal purposes. These have proven their high potential in medical applications: cardiac, vascular and orthopaedic conditions in adults and children. However, these require suitable mechanical properties and hygienic attributes in order to guarantee a low toxicity of degradation substances. Among products based on biodegradable materials, there are also different types of surgical sutures, orthopaedic hardware, vessel and bone implants, artificial skin, controlled medical substance dosing systems and drug stabilizing emulsions. Biomaterial is a latest development. It is a material unable to exist on its own but is employed in medicine to interact with biological systems. In the case of biological materials, their biocompatibility with tissues and blood (and its morphotic elements) is essential [25].

In the production process, starch serves as a filler known as thermoplastic starch (TPS). It is a component of synthetic polymers or is used as a basic raw material for the manufacture of a new type of biopolymers, such as polylactic acid (PLA) [26,27]. Polyvinyl alcohol is a functional additive widely used in the production of biopolymers. Its use in the production of biodegradable foamed materials goes back to the end of the 20th century; the technology itself, preceded by research, was designed by Neumann and Seib in 1993 [28]. The first foamed materials with the addition of polyvinyl alcohol were patented by Lacourse and Altieri in 1989 and 1991 [27,29,30]. 

The aim of the study was to assess the efficiency and energy consumption during the processing of starch-based foams with various polyvinyl alcohol (PVA) addition as well as measuring selected mechanical properties of foams as well as FTIR spectra depending on the processing conditions and forming dies used. 

## 2. Materials and Methods 

### 2.1. Raw Materials

Potato starch of the Superior Standard type (PPZ Trzemeszno, Trzemeszno, Poland) was used in the study. The moisture content of the starch was 16.7% and the pH 7.4. Polyvinyl alcohol PVA (Avantor-POCH S.A, Gliwice, Poland) was used as the foaming agent in the amount of 1%, 2% and 3% (w/w). PVA with the melting temperature 160–200 °C was odor-free white powder with the density of 1.2–1.3 g cm^−3^. Starch and PVA blends were moistened with water to 17%, 18% and 19% of the moisture content. To prepare the proper initial moisture, the amount of water was calculated per kg of dry mass as follows:

(1) $$x=\frac{M\times \left(W_{c}-W_{m}\right)}{100-W_{c}}\;\left(kg\right)$$

where: x—water amount (kg), M—mass of raw materials (kg), Wc—requested moisture content (%) and Wm—real moisture of raw materials (%).

### 2.2. Processing of Foams

**Figure 1:**
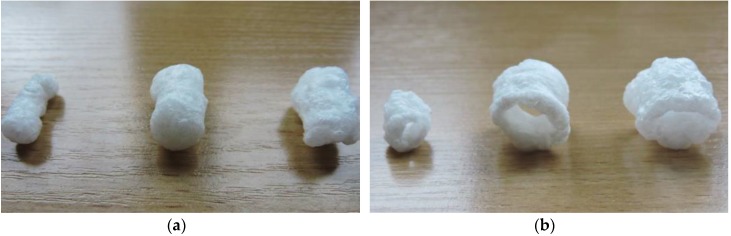
Obtained starch-based foams: (**a**) circular die and (**b**) ring die.

The blends were mixed for 20 min in a laboratory ribbon mixer. The TS-45 single screw extruder-cooker (Gliwice, Poland) was used to process the starch foams with the L/D ratio of 12. The foams were processed at the temperature below 120 °C with various screw speeds (100 and 130 rpm) and with two types of forming dies: circular (3 mm in diameter) and ring (external crevice of 5 mm; Figure 1). 

When composing raw material blends, the following symbols for individual tests were used: (a) M1—circular die, (b) M2—ring die, (c) S1—extruder-cooker screw speed 100 rpm, (d) S2—extruder-cooker screw speed 130 rpm, (e) A1—1% addition of polyvinyl alcohol, (f) A2—2% addition of polyvinyl alcohol and (g) A3—3% addition of polyvinyl alcohol

### 2.3. Evaluation of Process Efficiency

The efficiency of extrusion-cooking of starch-based foamed materials was determined by the sampling of foamed extrudates at a specific time. The efficiency was calculated according to the formula [31]:

(2) $$Q=\frac{m}{t}\;\left(kg\;h^{-1}\right)$$

where: Q—process efficiency (kg h^−1^), m—mass of extrudate obtained in the measurement (kg) and t—time of measurement (h). 

### 2.4. Specific Mechanical Energy Consumption

Energy consumption, expressed as SME (specific mechanical energy), was calculated taking into account the engine load, the extruder-cooker working parameters and the process efficiency of each test according to the formula [32,33,34].

(3) $$SME=\frac{n}{n_{m}}\times \frac{O}{100}\times \frac{P}{Q}\;\left(kWh\;kg^{-1}\right)$$

where: SME—specific mechanical energy consumption (kWh kg^−1^), n—screw rotation (rpm), nm—screw rating rotation (rpm,) O—engine load compared to maximum (%), P—rated power (kW) and Q—process efficiency (kg h^−1^).

### 2.5. Cutting Force 

**Figure 2:**
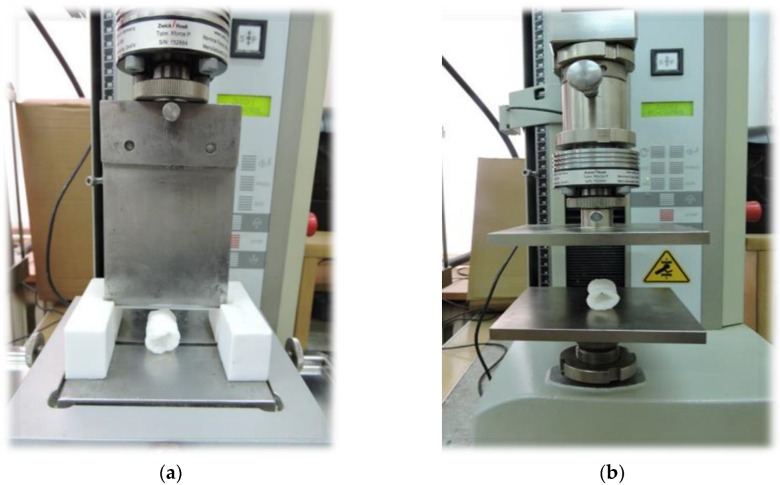
Zwick type BDO-FBO0.5TH equipped with: (**a**) Warner–Bratzler’s knife and (**b**) compression flat plates.

The cutting force and resistance to compression were evaluated using the Zwick BDO-FBO0.5TH universal testing machine (Zwick GmbH & Co. KG, Ulm, Germany). To determine the cutting force (N), Warner–Bratzler’s equipment (Figure 2a) was used according to the method described by Jin et al. [35], however, with some modifications. The samples were cut at the test speed of 100 mm min^−1^ for all blends and processing conditions. The samples (20 mm in length) were put on a working table at the angle of 90° to the cutting knife; the initial force was set to 0.1 N before recording the results. TestXpertII v3.3 software (ZwickRoell, Ulm, Germany) was used to record the results based on force-displacement curves. The mean value from 10 replications was the final value of the cutting force.

### 2.6. Compression Test 

The Zwick BDO-FBO0.5TH apparatus (Ulm, Germany) with two flat plates (Figure 2b) was used for the evaluation of resistance to compression. A compression test between two flat plates to 50% of the original height of the sample was carried out to determine resistance to compression (MPa) -presented as material elasticity. The test speed was set to 100 mm min^−1^, and the measurements were made in the transverse direction to the diameter of produced extrudates. The initial force was set to 0.1 N before recording the results. TestXpertII v3.3 software was used for the analysis of results from 10 replications, and the mean values were taken as the final result [36,37,38]. 

### 2.7. FTIR Analysis

Infrared spectra measurements for the analyzed samples were conducted with the 670-IR spectrometer (Agilent, Santa Clara, CA, USA). An ATR (attenuated total reflection) attachment was used in the form of a ZnSe crystal with adequate geometry (truncated at 45°) to ensure 20-fold internal reflection of the absorbed beam. During the measurement, 16 scans were registered and, subsequently, the program averaged the results for all spectra. Prior to the measurement, the ZnSe crystal was cleaned using ultra-clear solvents by Sigma-Aldrich. Prior to (1 h) and during the experiment, the measurement chamber was kept in an inert N_2_ atmosphere. Spectral measurements were recorded in the region from 500 to 4000 cm^−1^ at the resolution of 1 cm^−1^. The measurements were conducted at the Central Apparatus Laboratory of the University of Life Sciences in Lublin. The spectra were analyzed and processed with the Grams/AI software by ThermoGalactic Industries (Waltham, MA, USA). All the spectra were measured at 23 °C. 

### 2.8. Statistical Analysis 

The results obtained during the multiple tests were archived and statistically analyzed using the following software: Microsoft Excel 2014, SAS Enterprise Guide 5.1, and Statistica 13.3 (StatSoft, Cracow, Poland). The Excel software was used for calculating mean values with a standard deviation; the SAS Enterprise Guide 5.1 software was used for the multidirectional ANOVA analysis of variance with interactions; Tukey’s test was used for the means comparison at α = 0,05 level. For statistical analysis, RSM (response surface methodology) was used for fitting polynomial models; also - the quadratic equations of the tested characteristics were evaluated depending on the variables used in the experiment (Table 1).

### 2.9. Principal Component Analysis (PCA) of FTIR Spectra

The principal component analysis (PCA) was based on the data array of FTIR spectra of all examined samples. The first several scores of the PCA results were used to make a projection plot that provided a visual determination of similarities across the FTIR spectra. The OriginPro software (OriginLab, Northampton, MA, USA) was used for the PCA in this work.

## 3. Results and Discussion

### 3.1. Processing Efficiency of Starch-Based Foams

**Figure 3:**
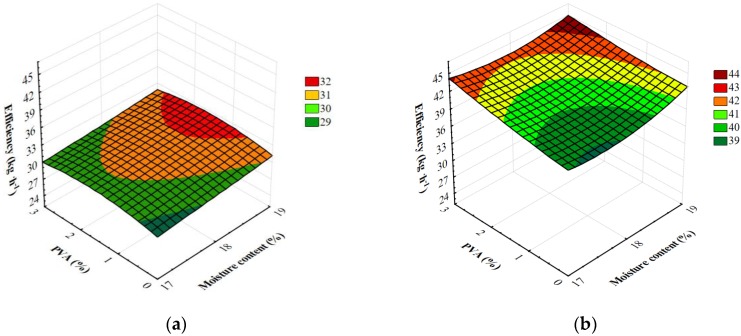

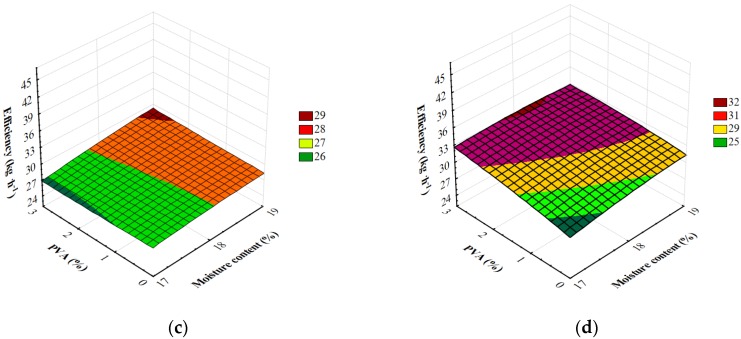
Efficiency of starch foams extrusion with various amounts of PVA and the initial moisture content of raw materials processed with different forming dies at various screw speed: (**a**) M1-S1, (**b**) M1-S2, (**c**) M2-S1 and (**d**) M2-S2.

The results of processing efficiency measurements are shown in Figure 3. These allow for different forming dies, screw speeds during extrusion, the level of moisture in the raw material blends and the amount of PVA used. The measurement results fell within 25.44–45.60 kg h^−1^.

The measurements of all variables adopted in the research showed a significant fluctuation in the efficiency of the extrusion-cooking process. Table 2 shows the results of a multifactorial analysis of variance addressing the research problem and demonstrate the influence of individual variables and their interactions on the efficiency of the extrusion-cooking process. The obtained degree of model matching with the experimental data revealed a high coefficient of determination R^2^ = 0.98. The critical level, given in the last column of Table 2, shows that all the variables adopted in the research and their double interactions modified the efficiency of barothermal treatment significantly. Additionally, based on the value of the F_0_ test, it must be underlined that the rotational speed of the extruder-cooker screw and the type of die used had a major effect on the efficiency of the extrusion-cooking of starchy foams. In addition, simultaneous comparisons showed an increase in the efficiency of extrusion-cooking along with a higher addition of polyvinyl alcohol at higher screw speeds during the production of starchy foams.

Changes were observed in the efficiency of extrusion-cooking of foamed starch materials depending on the level of moisture added to the raw material blends. As a result of the Tukey’s test and the observation of the extrusion-cooking process, significantly higher mean efficiencies of extrusion were reported for blends with a higher level of moisture. Based on obtained results of simultaneous comparisons, a significant decrease in the efficiency of the extrusion-cooking process was confirmed after reduction of the moisture level in the processed raw materials. The said efficiency was higher with a higher screw rotational speed for all raw material blends tested. A similar impact was observed in earlier studies [39] that studied the processing of potato and corn starch. In the course of the measurements, a higher extrusion efficiency was observed for blends processed using the M1 die; lower efficiency values, however, were reported for the processing of potato starch using the M2 ring die. Tukey’s test showed significantly higher mean extrusion efficiency for blends processed using the M1 die.

**Figure 4:**
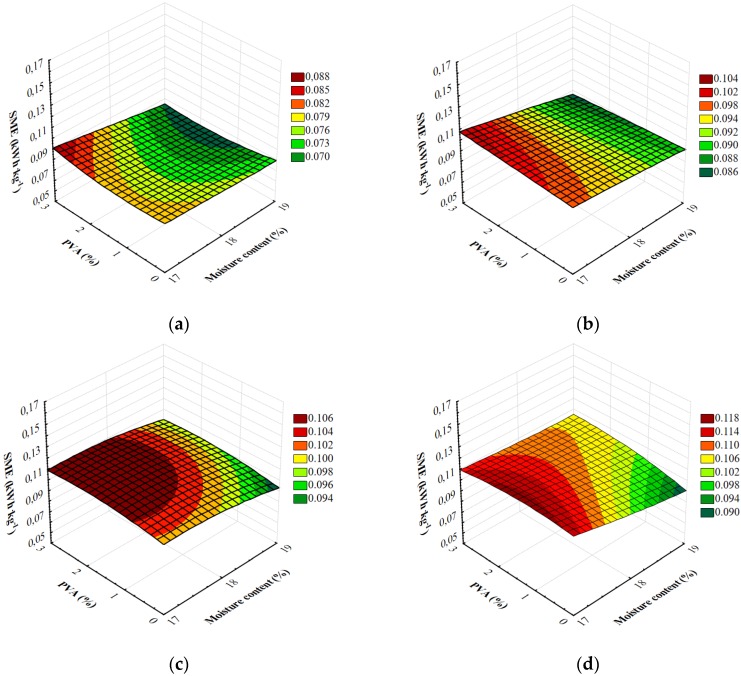
Specific mechanical energy of starch foams extrusion with various amounts of PVA and the initial moisture content of raw materials processed with different forming dies at various screw speeds: (**a**) M1-S1, (**b**) M1-S2, (**c**) M2-S1 and (**d**) M2-S2.

The results of SME measurements are shown in Figure 4. These allow for different forming dies, screw speeds during extrusion, the level of moisture added to the raw material mixture and the amount of PVA used. The results of the measurements ranged 0.070–0.121 kWh kg^−1^.

From the perspective of processing and industrial processes, the use of the extrusion-cooking technique in the production of foamed starch materials should have as low energy-intensity as possible. Energy-intensity of the extrusion-cooking process was determined based on the special mechanical energy values (SME). The results of measurements of all variables adopted in the research showed major variations in energy-intensity of the extrusion-cooking process. Table 3 shows the results of a multifactorial analysis of variance regarding the research problem and the influence of individual variables and their interaction on energy-intensity of the extrusion-cooking process. The degree of model matching with the experimental data was characterized by a high coefficient of determination R^2^ = 0.97. Based on the critical level given in the last column of Table 3, it was found that all the variables adopted in the study and their double interactions significantly differentiated the efficiency of extrusion-cooking treatment. Based on the value of the F_0_ test, it should be noted that the type of forming die used in the extrusion-cooking process and the level of moisture in the raw material blend had the strongest effect on energy-intensity of the extrusion-cooking process. 

The detailed comparisons of the means using Tukey’s test showed major discrepancies between all means for each variable except for the adjutant used in the study. In the case of 2% and 3% addition of polyvinyl alcohol (PVA), no significant differences were found between the means. The use of PVA in the production process influenced energy-intensity of the extrusion-cooking process. The higher amount of the additive, the higher the values of SME in the majority of raw materials blends. In addition, the results of simultaneous comparisons showed an increased energy consumption of the process along with a higher addition of adjutants.

During the measurement of energy-intensity of the extrusion-cooking process of foamed starch materials, the influence of the moisture content in the raw material blends on the SME value was also examined. For all the samples produced using the M1 die and the vast majority of blends processed using the M2 die, the higher level of moisture resulted in a reduced demand for mechanical energy. The same tendency was observed by Willett and Shogren [40], who extruded blends of starch and various thermoplastic resins into foams. Resins included poly(vinyl alcohol), cellulose acetate (CA) and several biodegradable polyesters. Nominal moisture contents ranged from 13.2% to 15.1% during extrusion. The results showed that with the increase in moisture SME value reduced, as the starch would be more highly plasticized by water [40].

After Tukey’s test, significantly higher energy-intensity of extrusion-cooking was reported for blends with a lower level of moisture. The results of simultaneous comparisons confirmed a significant decline of energy intensity of the extrusion-cooking process after raising the moisture level in the processed raw materials.

The rotational speed of the extruder-cooker screw influenced energy-intensity of the extrusion-cooking of foamed starch materials. The measurement results for the majority of raw material blends with the addition of PVA showed that the value of SME was higher at the higher screw speed. Oniszczuk et al. [21] and Mitrus and Mościcki [38] obtained similar results. Specific mechanical energy is an important factor in the extrusion of thermoplastic starch, as SME is related to the structural and functional properties of starch [41]. In general, SME is dependent on extrusion parameters such as screw speed, moisture content of the feed, temperature and mass flow rate. The progressive increase in SME with increasing screw speed is expected as these parameters are directly proportional to each other, provided torque in constant. This reflects a larger amount of mechanical work being performed on material in extruder. Summarizing, an increase in SME with increasing screw speed is typically observed in starch extrusion [41,42,43].

The completed Tukey’s test confirmed the existence of the relationship in question. A significantly higher mean energy intensity of the process was reported for the screw speed of 130 rpm. Higher energy consumption values of the extrusion-cooking of foamed starch materials were observed when using the M2 die for the processing of most raw material blends. Tukey’s test showed a significantly higher mean energy intensity of extrusion in blends processed using the M2 die.

**Figure 5:**
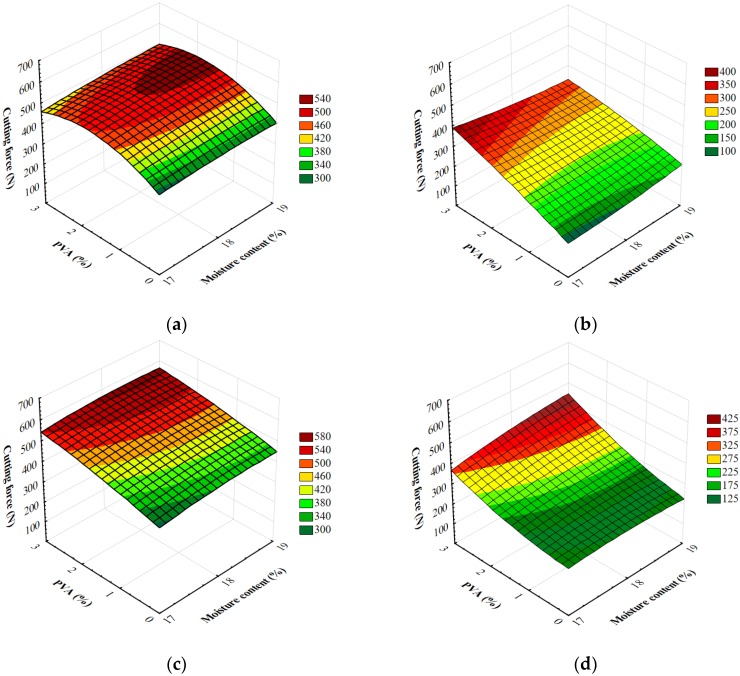
Cutting force of starch foams extrusion with various amounts of PVA and the initial moisture content of raw materials processed with different forming dies at various screw speed: (**a**) M1-S1, (**b**) M1-S2, (**c**) M2-S1 and (**d**) M2-S2.

To determine the cutting force of extruded starch foams is particularly important considering their potential functional use. The cutting force measurements are shown in Figure 5. These allow for different forming dies, screw speeds during extrusion, the level of moisture in the raw material blend and the amount of PVA used. The results fell within 102–642 N.

Measuring the strength of foams in a cutting test is important since such products are intended for functional applications, e.g., for the protection of items susceptible to mechanical damage. During the analysis of measurement results, and for all the variables studied in the research, a significant variation of the cutting force of starch foams was confirmed. Table 4 shows the results of the analysis of variance regarding the research problem and the influence of individual variables and their interactions on the cutting force of foamed extrudates. The degree of model matching with the experimental data was characterized by a relatively high coefficient of determination R^2^ = 0.80. The critical level *p* given in Table 4 indicates that the majority of the variables adopted for the study and the majority of their double interactions significantly differentiated the cutting power of foamed starch materials. Based on the value of the F_0_ test, the type of the die was thought to have the strongest effect on the tested feature.

As a result of the detailed mean comparisons in the Tukey’s test, significant differences were exposed between all the mean cutting forces only for the type of the forming die. Between some means reported for the tested additives and moisture levels, the Tukey’s test simultaneous comparisons showed a significant variation. In the case of 2% and 3% addition of polyvinyl alcohol, 17% and 18% of moisture and the two screw rotational speeds, no significant differences were found between the means. In the case of polyvinyl alcohol, when increasing the quantity of the additive, the value of the tested parameter was rising, too. Based on the completed Tukey’s test, higher mean cutting forces of blends with polyvinyl alcohol were reported than in the case of control samples. Superior cutting force values were seen in foams obtained from the raw material blends with a higher moisture level. The highest cutting force was reported in products made from blends with the highest moisture level (19%). Completed Tukey’s test confirmed the relationship in question. Measurements for the majority of foams produced at higher rotational screw speeds showed lower values of the tested feature. A higher cutting force was noted for samples produced at the screw speed of 100 rpm. Superior cutting force values were recorded in all samples produced with the M2 die. A significantly lower mean cutting force was seen in extrudates produced with the M1 die.

**Figure 6:**
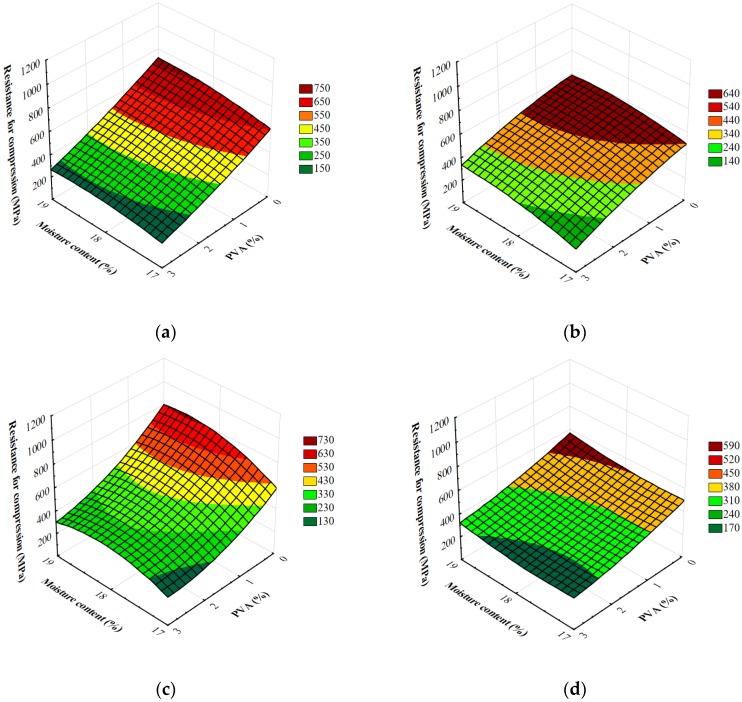
Resistance for the compression of starch foams extrusion with various amounts of PVA and the initial moisture content of raw materials processed with different forming dies at various screw speed: (**a**) M1-S1, (**b**) M1-S2, (**c**) M2-S1 and (**d**) M2-S2.

A compression test, as follows from the definition, is used for brittle, easily damaged materials. However, also softer materials (less likely to crack during compression) undergo this test to measure their flattening when high force is applied. The compression process involves two basic values: deformation and stress. Similar values are vital in stretching. When the elasticity limit is exceeded, permanent deformation in the tested sample is observed. The plastic material swells and takes a barrel-like shape. Consequently, the diameter of the sample increases and, as a result, more force is needed to continue deformation. The test is usually considered completed when clear plastic deformation is observed. In the case of brittle materials, samples are destroyed already after small deformations. The so-called sample chipping occurs. Owing to the compression test of plastics, it is possible to describe the full functional characteristics of the material. The measurements of resistance to compression are shown in Figure 6. These allow for different forming dies, screw speeds during extrusion, the level of moisture in the raw material blend and the amount of PVA used. The results varied and ranged from 133 to 935 MPa. For all the variables adopted in the research, the results showed major variations in stiffness of foamed starch materials. Table 5 shows the results of the analysis of variance regarding the research problem and demonstrates the influence of individual variables and their interaction on the stiffness of extruded foams.

The degree of model matching with the experimental data was characterized by a relatively high coefficient of determination R^2^ = 0.76. The critical level, given in the last column of Table 5, shows that all the variables adopted in the research and the vast majority their double interactions differentiated the tested feature. Additionally, based on the F_0_ test, the amount of additive was considered to have the strongest effect on stiffness of produced foamed extrudates. The control samples showed the highest stiffness. After the use of adjutants, in the majority of raw material blends the value of the tested feature decreased. The higher the PVA additive, the lower stiffness of obtained products. Indeed, the highest average stiffness was reported for blends without adjutants added. Significantly higher mean values of the tested feature were reported for the majority of samples with the addition of PVA. The level of moisture in the raw material blends had an effect on the stiffness of foamed starch materials produced during the extrusion-cooking process. The research showed higher values of the tested feature for foams made from raw materials with higher moisture content. The highest mean stiffness was observed in foams made from blends with the highest moisture level. Based on the results of simultaneous comparisons, a significant decrease in stiffness of foamed starch was confirmed after the reduction of the moisture level in the processed raw materials. The results of stiffness tests of foamed extrudates reported during the compression test showed the influence of the screw rotational speed on the value of the tested feature. As the screw speed increased, stiffness of foamed starch materials decreased. Significantly higher mean stiffness was seen in the extrudates produced at the screw speed of 100 rpm. During the measurements, stiffness was observed to be influenced by the shape of the die. Higher values of the tested feature were obtained for all samples made on the M1 die. According to Mitrus [6], some mechanical properties of foamed starch materials (including stiffness) are comparable to those of commercial polymer foams. Stiffness of the extrudates undergoing the compression test ranged from 53.5 to 1120 MPa. The obtained values were higher than those found in the literature on compression tests of extrudates based on maize starch [38].

### 3.2. FTIR Characteristics of Foamed Starch-Based Materials

Infrared spectra (FTIR) measurements are more and more frequently used for the analysis of various types of compounds, including materials containing biodegradable additives (thermoplastic starch, potato starch, technical glycerine, polyvinyl alcohol (PVA), polylactic acid (PLA), keratin hydrolysate and many more [44]), as well as for their determination in various types of products affecting their functionality. The capacity of using FTIR spectroscopy for the determination of the compounds listed above in biodegradable materials is demonstrated in more and more research works. This is due to the fact that, for example, the absorption of many functional groups, such as the carbonyl group C=O (1700–1800 cm^−1^, occurring in this type of additives either because of their degradation effects or introduced with additives used) in the starch fraction of the tested product is relatively scarce or is not present at all. On the other hand, absorption in this area (for the carbonyl group) quickly grows in intensity in materials containing various types of additives. It is also worth noting that starch alone produces very intense and interesting infrared spectra because, as one of the carbohydrates (plant polysaccharides), it consists of glucose units linked by *α*-glycosidic bonds (as mentioned earlier). However, starch actually consists of two main fractions: first of all, unbranched amylose made up of glucose residues connected by oxygen atoms with *α*-1,4-glycosidic bonds and, secondly, branched amylopectin with extra *α-1,6*-glycosidic bonds. The use of modifications by the extrusion-cooking in the tested samples also revealed substantial changes in the infrared spectra. These changes clearly suggest a significant modification of molecular interactions in the obtained group of two types of samples, which was reflected in modified FTIR spectra in materials selected for the research [45].

**Figure 7:**
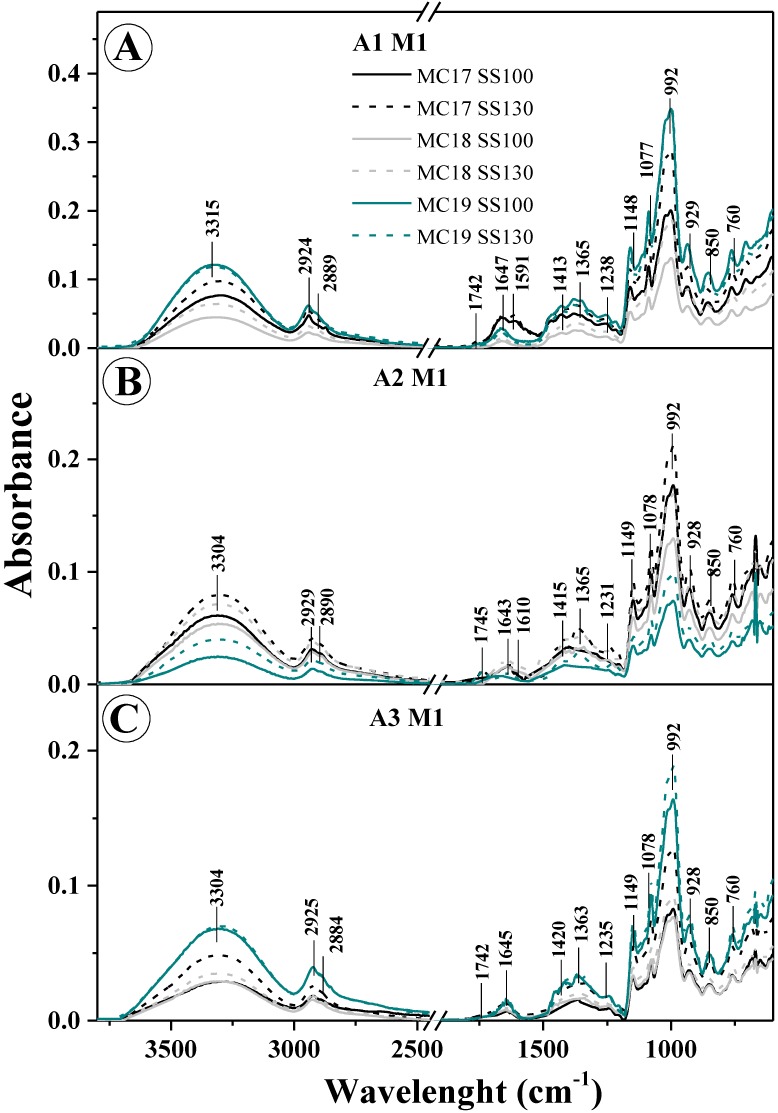
M1 extrudate with different PVA content, panel (**A**)—A1, panel (**B**)—A2 and panel (**C**)—A3.

For easier analysis and comparison of collected data, obtained FTIR spectra are shown in Figure 7. On the other hand, Table 6 presents all vibrations occurring in the tested samples (in both types of forms) with their linking to vibration spectra of specific functional groups [46,47].

It should also be noted that only M1 samples were selected for the FTIR and PCA analysis (discussed below) because of the extensive volume of data. Still, these are sufficient and representative to discuss obtained results. It is evident that, due to the use of appropriate modifications/additives (such as variable humidity MC17, 18, 19, screw speed (100 or 130) or the addition of PVA), the structure of the samples selected for testing underwent visible changed, as demonstrated in the obtained infrared spectra. Such noticeable changes, mainly in intensity (but also in the structure of the spectra), clearly point to strong intermolecular interactions in the studied starch systems. Clearly, some water content can be observed in all the tested samples, as evidenced by the presence of quite intense spectra in the range of around 1640–1647 cm^−1^. These are deformation vibrations of the groups -OH - δ_m_(OH) (absorber water) and from the range of 3600-3000 cm^−1^ with a peak at 3200 cm^−1^ and belong to stretching vibrations OH, ν(-OH) with absorber water. A change in intensity of these spectra obviously depends on the level of moisture used to obtain the given sample, however, interestingly, in several cases, it also depends on the extruder-cooker screw speed (Figure 7). Vibrations with a peak at about 1640 cm^−1^ may partly overlap with vibrations belonging to the material of the tested samples. However, vibrations ranging 3600–3000 cm^−1^, which is the standard range of stretching vibrations of groups -OH, can be generated by free (intra- and extramolecular) hydrogen bonds in the structure of starch used to obtain the tested samples. Along with the change of additives used with the tested samples, the formation of hydrogen bonds may modify the internal structure of samples (and thus significantly modify the spectra observed). Another noteworthy vibrations range is 3000–2800 (also characteristic of this kind of material of natural origin), which indicates C-H stretching vibrations belonging to CH_2_ groups. The vibrations described earlier with a peak of about 1640 cm^−1^ (deformation –OH) also originate in the vibrations of water molecules that are bound within the starch structure. In turn, the vibration range of 1300–1100 cm^−1^ is occupied by the stretching asymmetric C-O vibrations and C-O-C group vibrations. These vibrations typify molecule systems in natural polysaccharides. Next, a very important range of 930–700 cm^−1^ is assigned to vibrations characteristic of the polysaccharide ring. These belong to the pyranose ring in the individual units belonging to glucose itself.

For the range of 3600–3000 cm^−1^ (Figure 7), the characteristic wide spectrum corresponding to the stretching vibrations of the –OH group showed variable intensity primarily depending on the moisture level of the given sample (regardless of its form) but also because of the rotational speed applied in their production. In addition, in the majority of the samples obtained, the peak of this spectrum was significantly transferred towards larger wave numbers, which clearly suggests the formation of (multiple) intermolecular hydrogen bonds. This is quite important because it implies a significant impact of the screw speed on the quality of obtained products, i.e., on the homogeneity of the final material from production. This fact is also confirmed by the greater/variable intensity of the spectrum associated with deformation vibrations –OH (with a peak of 1640 cm^−1^), associated with the vibrations of particles in starch itself. For vibrations in the range of 3000–2800 cm^−1^ (characteristic of stretching vibrations -CH_2_, described earlier), the most intense spectra are observed in samples that exhibit changes in the ranges characteristic of various types of vibrations associated with water molecules. This may be related, first of all, to a similar content of amylose and amylopectin in the tested material and with a greater strength of intermolecular interactions in the tested samples when applying specific modifications during their production (like a variable amount of PVA additive). In the FTIR spectra of the examined forms altered with all additives/modifications, very deep changes were observed (mainly in spectra intensity) with a peak at about 1250–70, 1370 and for the range of 950–1190 cm^−1^. These vibrations are associated with the skeletal ones of C-O and C-C groups, characteristic of the starch used to produce the samples. First, this may testify to the very good homogeneity of ingredients used for the production of the mixtures/forms, and, second, to the existence of strong molecular interactions between them through the creation of strong hydrogen bonds between individual groups. Below about 850 cm^−1^, there are spectra characteristic of *α*-glycosidic bond, i.e., typical of starch only.

It is worth noting that changes observed in this area, especially along with the increase in the amount of PVA additive (practically, in all implemented modifications), prove structural changes within the glycosidic bond itself, but, of course, these depend on the moisture level of the samples. 

To sum up, it is evident that the additives/modifications used significantly modify the FTIR spectrum of both types of obtained forms [46,47]. It proves a major impact of the additive, both in starch itself as well as between the starch molecules and the additive used (PVA), and the variable humidity level on intermolecular interactions, which was also confirmed by Kizil et al. [46].

### 3.3. PCA of FTIR of M1 Sample with PVA Addition 

In order to pinpoint the relationship between the investigated FTIR spectra and PVA addition, as well as selected mechanical properties of foams depending on the processing conditions and forming dies used, the PCA method was applied.

The use of mathematical and statistical methods, including chemometrics and many other statistical analysis and mathematical algorithms, in material science and technology has been on the constant rise in the last decades. There is a trend of combining these methods with spectroscopy, especially with FTIR spectroscopy, which is one of the most cost-saving and, still, very effective methods [48]. 

One of the most popular chemometric methods in material research is the principal component analysis (PCA). PCA is a method that isolates spectral information based on the variance maximum principle. The method involves the reduction of variables into new, low-dimension variables instead of the original high-dimension ones. This study shows the data of FTIR spectra [49]. 

**Figure 8:**
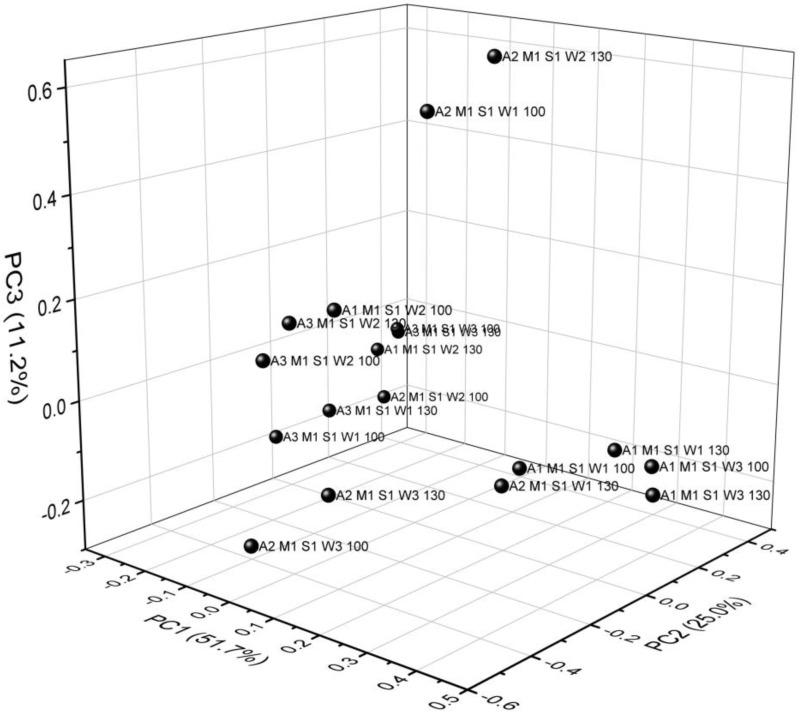
The scattered scores plot (PC1 × PC2 × PC3) based on FTIR spectra of M1 with various PVA addition.

As shown in Figure 8, a three-dimensional scatter plot with the principal components PC1, PC2 and PC3 was obtained from the FTIR spectra of M1 samples with various PVA amounts after the PCA analysis.

The first principal component was the most important factor, and its variance contribution ratio was 51.7%; the second principal component had a variance contribution rate of 25%, and the third one a variance contribution rate of 11.2%. The first three main components had a variance contribution ratio of 87.9%, and only 12.1% of the information was lost. This means that the first three principal components conveyed 87.9% of the all information. Therefore, the first three principal components actually reflected the vast majority of spectrum information.

### 3.4. Loading Analysis of FTIR Spectra Samples M1 with Different PVA Content

**Figure 9:**
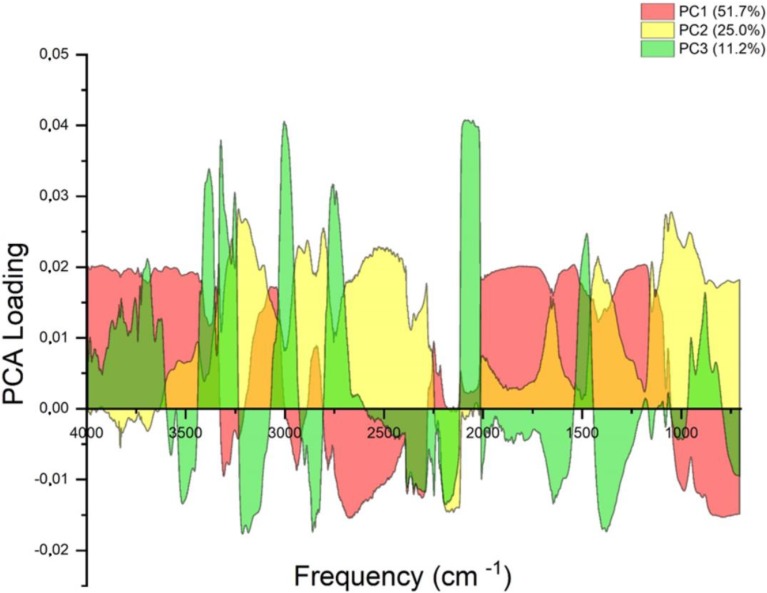
The loading factors of the principal component analysis (PCA).

More information concerning the basic differences of sample compounds can be obtained when using the PCA loading factors model. The loading factors atlas was drawn across the whole wave spectrum (Figure 9). PC1, PC2 and PC3 were the key factors in the PCA model with the high contribution rate of PC1 amounting to 51.7%. It means that PC1, PC2 and PC3 were sufficient to illustrate discrepancies in the composition of different samples of starch foams with various amounts of PVA [50,51].

Peaks with the lowest contribution rate to PC1 ranged from 757 to 928 cm^−1^ (Figure 9). This range was closely related to the type of vibrations as ν -stretching (C-C) and ν (C-O) or C-O-C bend or O-H deformation (intensified by water), as noted before (Table 6). The next two ranges were connected with ν_st_(C-H) and ν(C-H) in the CH_2_/CH_3_ group (2887 cm^−1^, 2925 cm^−1^) type of vibration and ν(-OH) with absorber water (3308 cm^−1^). These types of vibrations corresponded to the positive values of PC2. Peaks with a higher contribution rate to PC3 cover peaks that corresponded to the lowest contribution to PC2 rate. These results indicate that the differences among tested samples were mainly reflected in the different shape of asymmetrical from 1645 cm^−1^–2925 cm^−1^. This result matched that of the FTIR analysis.

## 4. Conclusions

All process variables had some influence on the efficiency and specific mechanical energy of the extrusion-cooking process. With higher addition of polyvinyl alcohol, the efficiency of extrusion-cooking process increased. For the majority of processed mixtures, higher moisture generated a higher value of efficiency. The efficiency of the process was higher with higher screw rotations for the tested mixtures. Higher efficiency was also observed for blends processed using the circular die. With an increasing amount of polyvinyl alcohol, SME of the extrusion-cooking process was higher. Energy consumption decreased with a higher level of moisture content in the blends. A higher screw speed led to higher energy consumption values. Higher SME of the extrusion-cooking process was obtained during blend processing using the ring forming die. The results showed that the processing efficiency of potato starch foams varied depending on the level of polyvinyl alcohol, the shape of the forming die and the screw speed applied. A statistical analysis, including the F-test, demonstrated that the screw speed and the shape of the forming die were the most significant factors in the processing efficiency of the tested foams. The analysis of SME results proved that the type of the forming die and the initial moisture level had the most significant effect on specific mechanical energy requirements during the processing of potato starch foams. The samples obtained during the direct extrusion process were characterized by variable mechanical properties. Increased addition of polyvinyl alcohol to the recipe increased the cutting force but, at the same time, decreased stiffness of the tested foams. The F-test showed that the most significant effect of the forming die used in experiments was on cutting force values. However, as regards resistance to compression, the most significant effect was visible across variable levels of the additive.

As demonstrated, the use of FTIR analysis combined with chemometrics methods to investigate starch foams exposed the reasons for their varying compositions. The results obtained in this study could serve as a method of optimizing evaluation procedures for the purposes of quality control analysis of biodegradable packaging materials.

## Figures and Tables

**Table 1:** Adequacy of two-variable model fit to tested characteristics of starch-based foams.

Parameter	Forming Die	Screw Speed (rpm)	Model Fitted
Efficiency (kg h^−1^)	M1	100	E = 41.118−2.119MC+1.510A+0.084MC^2^+0.005MCA−0.464A^2^
		130	E = 436.168−45.329MC+6.344A+1.296MC^2^−0.317MCA+0.136A^2^
	M2	100	E = −48.549+7.922MC-3.726A-.204MC^2^+0.206MCA+0.036A^2^
		130	E = −97.566+13.166MC+7.026A−0.336MC^2^−0.322MCA−0.136A^2^
SME (kWh kg^−1^)	M1	100	SME = 0.2007−0.0103MC+0.0271A+0.0002MC^2^−0.0018MCA+0.0016A^2^
		130	SME = −0.0154+0,0173MC+0.0368A−0.0006MC^2^−0.002MCA−0.0001A^2^
	M2	100	SME = −1.0122+0.1293MC+0.0002A−0.0037MC^2^+0.0004MCA−0.0016A^2^
		130	SME = 0.6545−0.051MC−0.0427A+0.0011MC^2^+0.0028MCA−0.0016A^2^
Cutting force (N)	M1	100	CF = −2028.02+263.12MC+53.84A-7.11MC^2^+3.87MCA−32.28A^2^
		130	CF = 2860.28−319.26MC+384.05A+9.46MC^2^−17.41MCA−4.60A^2^
	M2	100	CF = −3592.86+430.68MC+92.52A−11.48MC^2^-2.02MCA−5.14A^2^
		130	CF = -3275.19+392.57MC−229.01A-10.96MC^2^+14.09MCA+11.72A^2^
Resistance for compression (MPa)	M1	100	RC = −9553.83+1079.09MC+43.37A-28.33MC^2^−11.76MCA+9.15A^2^
		130	RC = −11425.17+1289.41MC−187.74A−34.59MC^2^+7.64MCA−14.04A^2^
	M2	100	RC = -30643.98+3380.90MC+242.65A-90.84MC^2^−27.26MCA+36.21A^2^
		130	RC = 8347.24−906.55MC−58.97A+26.16MC^2^−3.08MCA+11.93A^2^

M1—circular die; M2—ring die; MC—moisture content; A—additive level.

**Table 2:** Statistical analysis of the effect of variation source and its interactions on starch-based foams processing efficiency (E).

Variation Source	df	Sum of Squares	Mean Squares	F_0_ Test	*p* (F > F_0_)
A	6	637.08	106.18	162.05	<0.05
M	1	9786.55	9786.55	14936.30	<0.05
MC	2	887.46	443.73	677.22	<0.05
S	1	18315.90	18315.90	27953.90	<0.05
A*M	6	162.12	27.02	41.24	<0.05
A*MC	12	395.04	32.92	50.24	<0.05
A*S	6	511.23	85.20	130.04	<0.05
M*MC	2	7.71	3.85	5.88	<0.05
M*S	1	454.97	454.97	694.38	<0.05
MC*S	2	9.09	4.55	6.94	<0.05

A—additive level; M—forming die; MC—moisture content; S—screw speed; df—degrees of freedom; *p*—significance value.

**Table 3:** Statistical analysis of the effect of variation source and its interactions on starch-based foams specific energy consumption (SME) during extrusion.

Variation Source	df	Sum of Squares	Mean Squares	F_0_ test	*p* (F > F_0_)
A	6	0.0042	0.0007	135.31	<0.05
M	1	0.1026	0.1026	19669.70	<0.05
MC	2	0.0308	0.0154	2955.75	<0.05
S	1	0.0068	0.0068	1297.34	<0.05
A*M	6	0.0285	0.0028	54.88	<0.05
A*MC	12	0.0017	0.0001	56.09	<0.05
A*S	6	0.0035	0.0003	99.17	<0.05
M*MC	2	0.0025	0.0001	30.77	<0.05
M*S	1	0.0001	0.0001	331.60	<0.05
MC*SS	2	0.0002	0.0001	16.30	<0.05

A—additive level; M—forming die; MC—moisture content; S—screw speed; df—degrees of freedom; *p*—significance value.

**Table 4:** Statistical analysis of the effect of variation source and its interactions on the cutting force (CF) of starch-based foams.

Variation Source	df	Sum of Squares	Mean Squares	F_0_ test	*p* (F > F_0_)
A	6	20835591.86	3472598.64	842.40	<0.05
M	1	16537802.65	16537802.65	4011.82	<0.05
MC	2	44607.10	22303.55	5.41	<0.05
S	1	398.41	398.41	0.10	0.76
A*M	6	1382125.77	230354.29	55.88	<0.05
A*MC	12	328018.80	27334.90	6.63	<0.05
A*S	6	805109.31	134184.88	32.55	<0.05
M*MC	2	21191.76	10595.88	2.57	0.08
M*S	1	212836.72	212836.72	51.63	<0.05
MC*S	2	42746.38	21373.19	5.18	<0.05

A—additive level; M—forming die; MC—moisture content; S—screw speed; df—degrees of freedom; *p*—significance value.

**Table 5:** Statistical analysis of the effect of variation source and its interactions on resistance for the compression (RC) of starch-based foams.

Variation Source	df	Sum of Squares	Mean Squares	F_0_ test	*p* (F > F_0_)
A	6	44380113.23	7396685.54	976.75	<0.05
M	1	1722479.66	1722479.66	227.46	<0.05
MC	2	1864091.80	932045.90	123.08	<0.05
S	1	2489633.17	2489633.17	328.76	<0.05
A*M	6	1276493.15	212748.86	28.09	<0.05
A*MC	12	498540.48	41545.04	5.49	<0.05
A*S	6	810771.30	135128.55	17.84	<0.05
M*MC	2	148377.25	74188.63	9.8	<0.05
M*S	1	678894.86	678894.86	89.65	<0.05
MC*S	2	164722.48	82361.24	10.88	<0.05

A—additive level; M—forming die; MC—moisture content; S—screw speed; df—degrees of freedom; *p*—significance value.

**Table 6:** Location of peaks of FTIR absorption spectra with the corresponding vibrations for materials selected for tests (biodegradable) over the 3800–550 cm^−1^ spectrum range.

Position o Maximum (cm^−1^)								
M1 S1	M2 S1	M1 S1			M2 S1			Type of Vibrations
		A1	A2	A3	A1	A2	A3	
3308	3308	3315	3304	3304	3317	3307	3300	ν(-OH) with absorber water
2925	2925	2924	2929	2925	2927	2927	2926	ν_st_(C-H) and ν(C-H) in CH_2_ / CH_3_ group
2887	2887	2889	2890	2884	2887	2886	2885	
1742/5	1742/5	1742/5	1742/5	1742/5	1742/5	1742/5	1742/5	ν(C=O)
1647	1645	1647	1643	1645	1645	1647	1640	δ_m_(O-H) (absorber water)
nd	nd	1591	1610	nd	nd	1609	Nd	δ(C-H) or δ(CH2) in plane
1417	1418	1413	1415	1420	1417	1417	1415	C-H bending and wagging or δ(COH)
1363	1363	1365	1365	1363	1367	1360	1363	
1241	1239	1238	1231	1235	1240	1241	1242	δ(O-H) or C-O
1148	1147	1148	1149	1149	1149	1147	1147	anhydroglucose ring C–O stretch of C–O–H in starch and C-O-C antisymmetric bridge
1077	1078	1077	1078	1078	1078	1078	1078	
990	990	992	992	992	993	992	992	ν (C-O) and ν(C-O-C or C-O-H)
927	927	929	928	928	927	928	928	ν (C-C) and ν (C-O) or C-O-C bend or O-H deformation (broadened by water)
850	850	850	850	850	850	850	850	
757	760	760	760	760	760	760	760	
757	760	760	760	760	760	760	760	

ν—stretching, δ—deformation, s—symmetric, as –asymmetric, st—strong, m—medium, nd—no data.
